# Supplementary figures and images for: Preservation of cellular nano-architecture by the process of chemical fixation for nanopathology
Source: PLoS One. 2019 Jul 22;14(7):e0219006. doi: 10.1371/journal.pone.0219006 (PMC6645510; doi:10.1371/journal.pone.0219006)

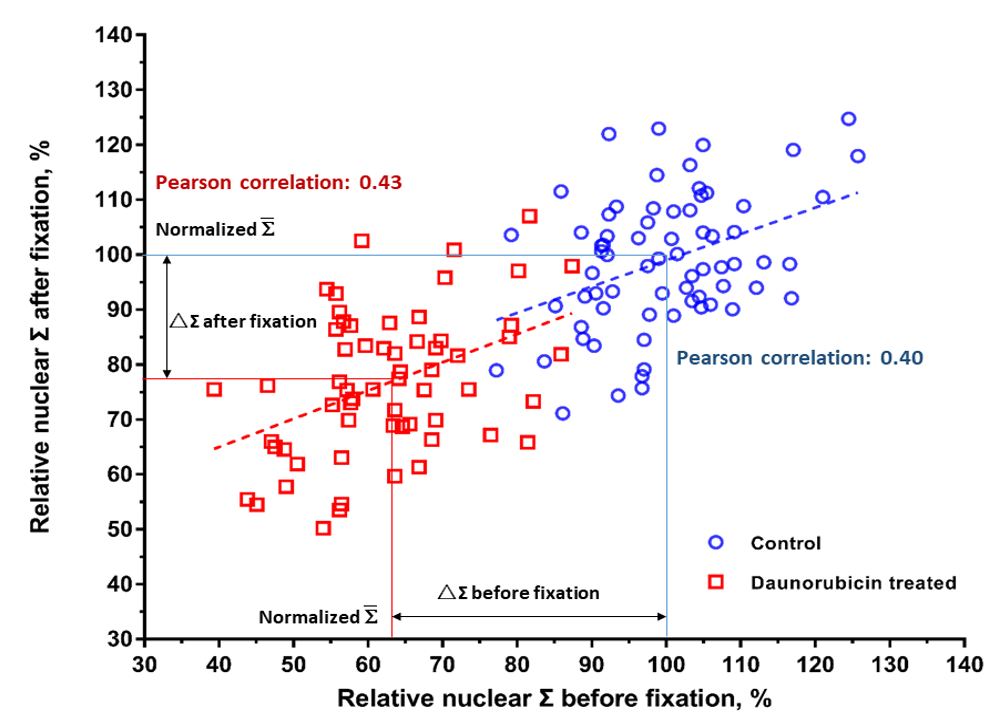

Supplement: S1 Fig — The same cells were tracked and imaged before and after 95% ethanol fixation (HeLa-control = 71 cells, HeLa-daunorubicin = 61 cells). The relative nuclear Σ was weakly correlated, but the population difference was preserved. (TIF) [file pone.0219006.s001.tif]

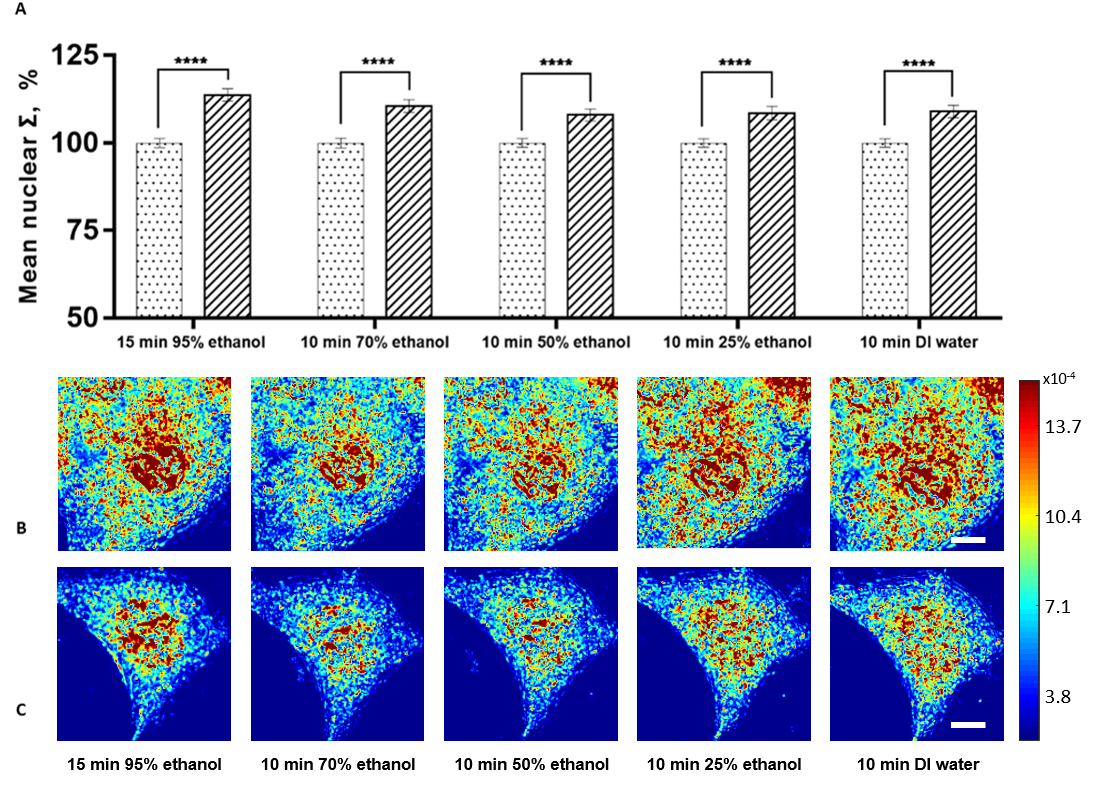

Supplement: S2 Fig — Quantification of Σ in (A) M248 (columns with dots; 61 cells) and A2780 (columns with stripes; 67 cells) after each step of serial rehydration with SE bars. The ΔΣ was preserved during serial rehydration in this models. Representative PWS images of the same cells [M248 (B) and A2780 (C)] after each step of serial rehydration. All scale bars are 8 μm. **** p<0.001. (TIF) [file pone.0219006.s002.tif]

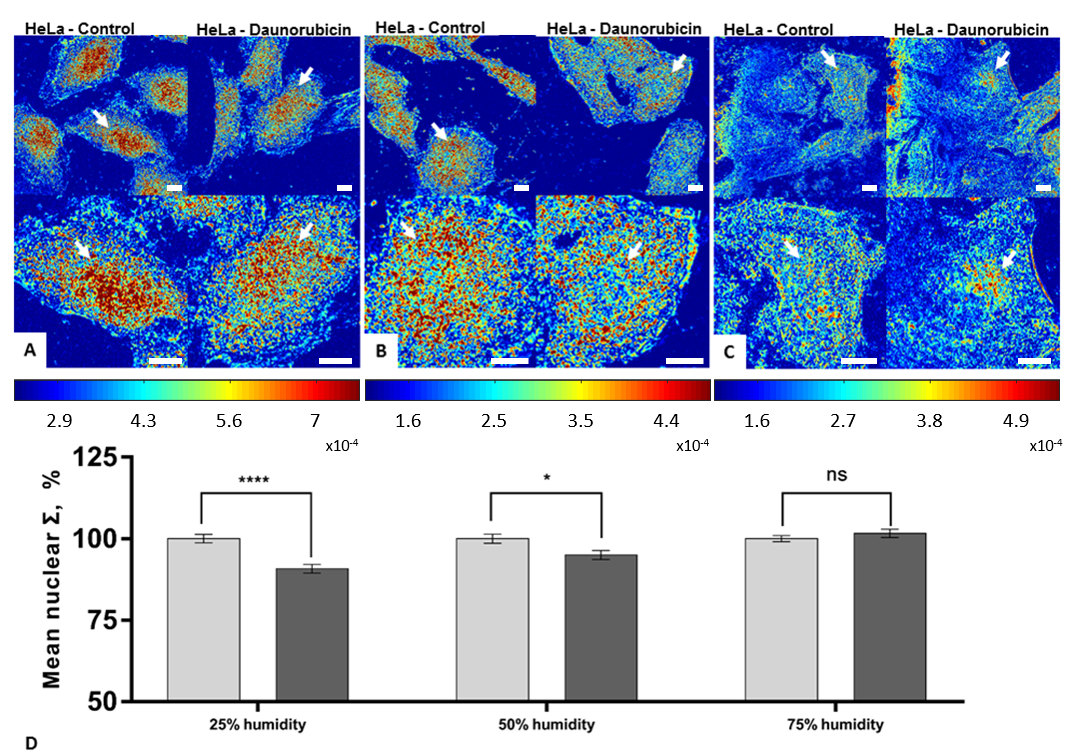

Supplement: S3 Fig — Representative images of HeLa model air dried in (A) 25% (±5%), (B) 50% (±5%) and (C) 75% (±5%) humidity. (D) Quantification of mean nuclear Σ (25% humidity: HeLa-control = 67 cells, HeLa-daunorubicin = 72 cells; 50% humidity: HeLa-control = 131 cells, HeLa-daunorubicin = 141 cells; 75% humidity: HeLa-control = 135 cells, HeLa-daunorubicin = 126 cells) with SE bars. All scale bars are 11 μm. **** p<0.001, * p = 0.01. (TIF) [file pone.0219006.s003.tif]

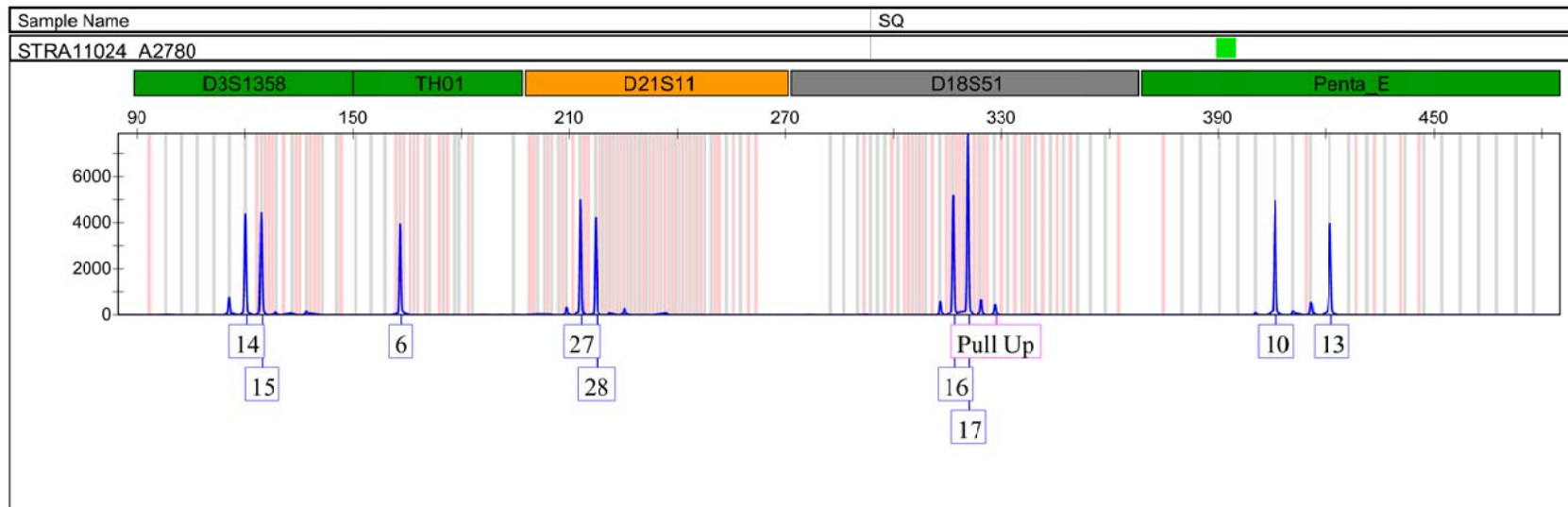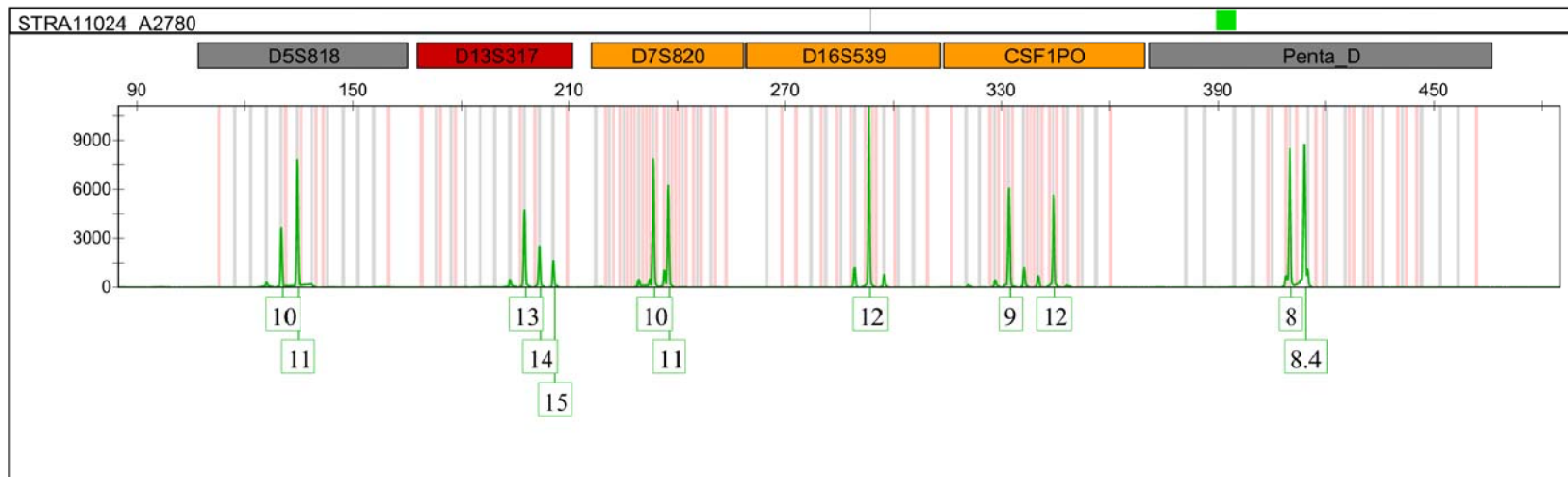

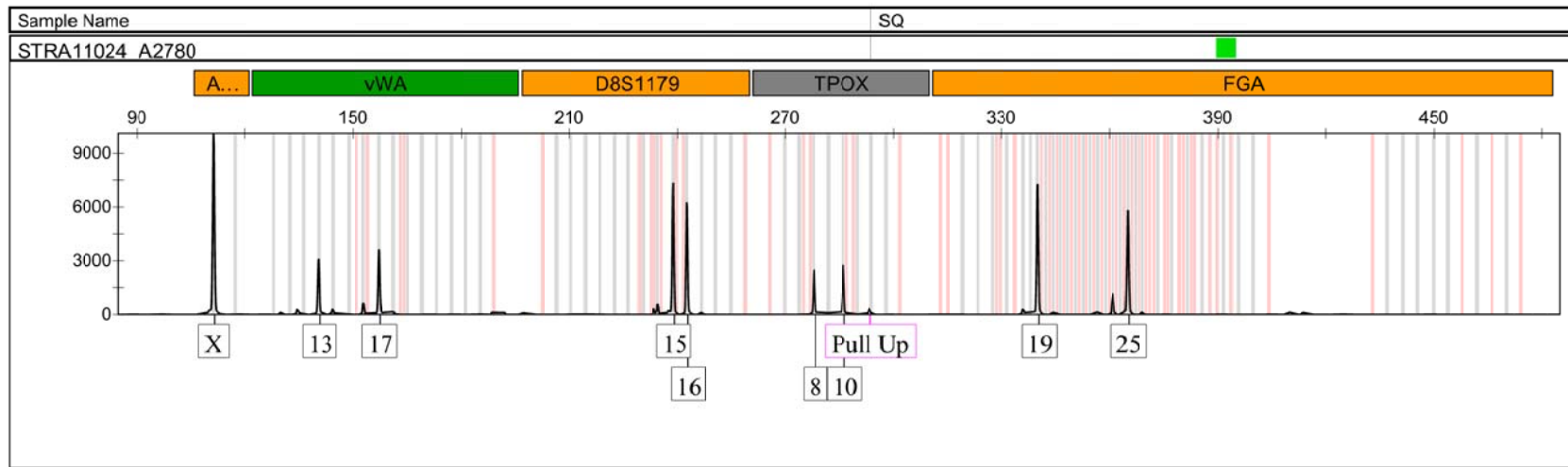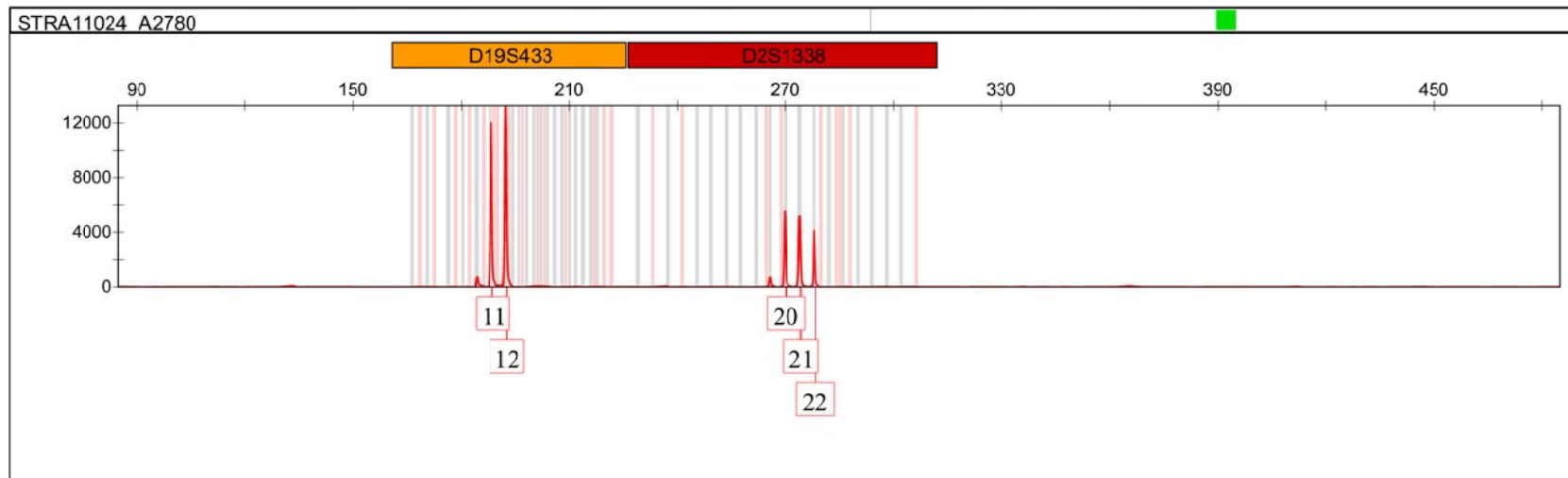

Supplement: S2 File — Short Tandem Repeat (STR) analysis of the A2780 derived cell line used in these studies. (PDF) [file pone.0219006.s005.pdf]

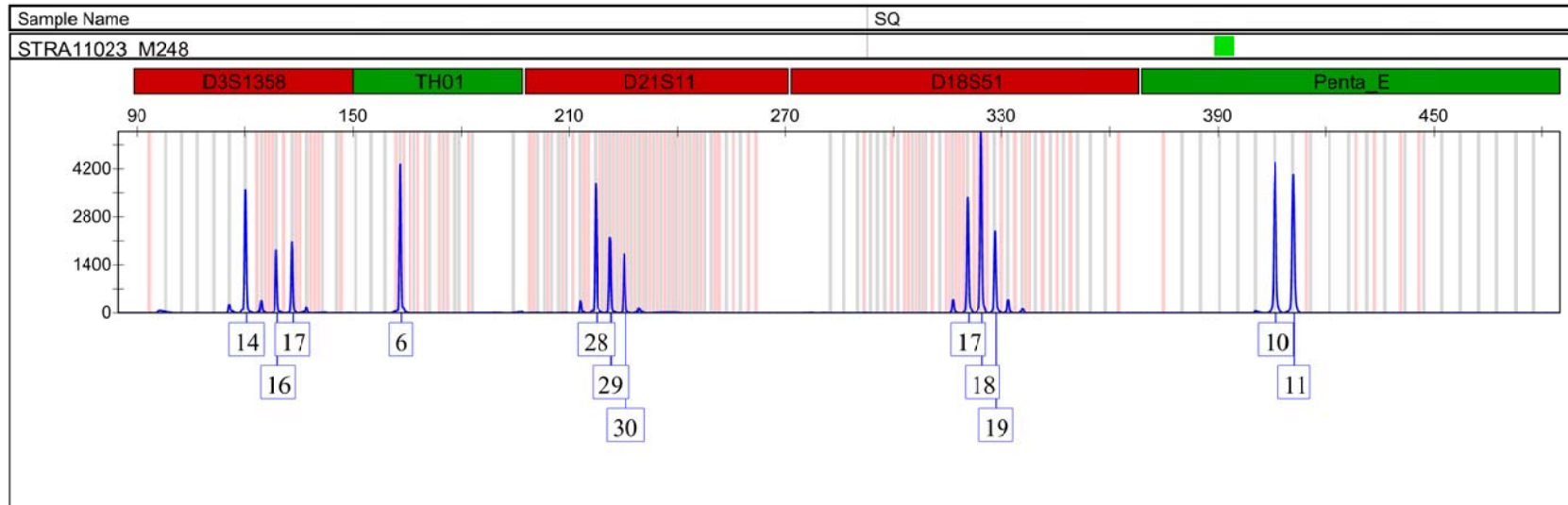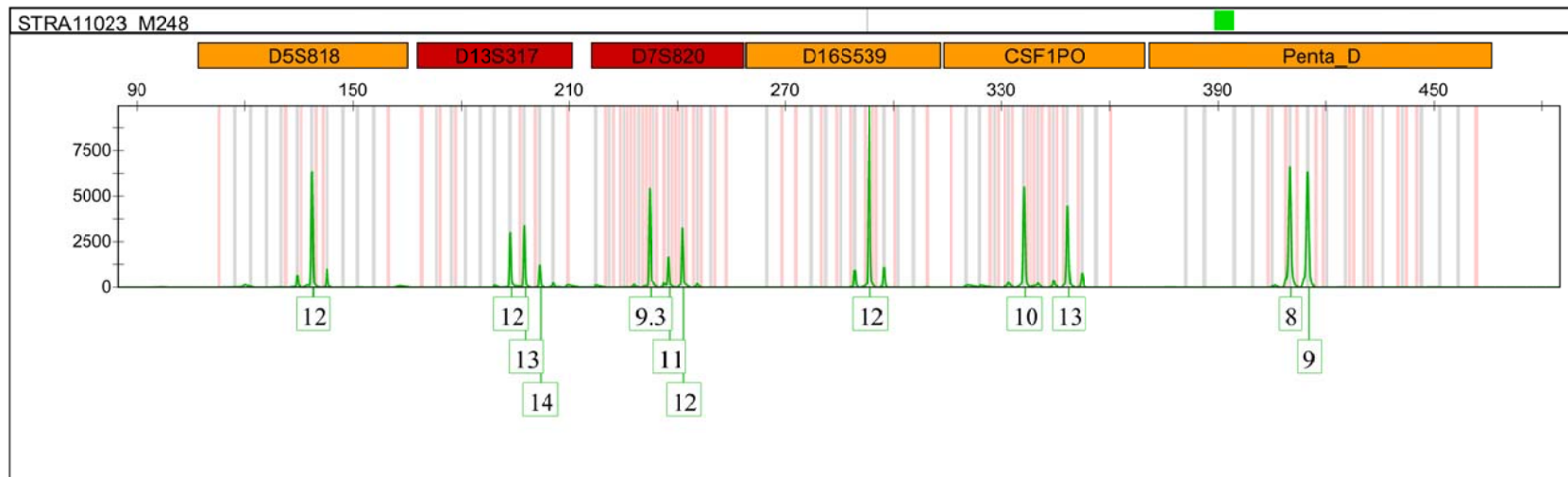

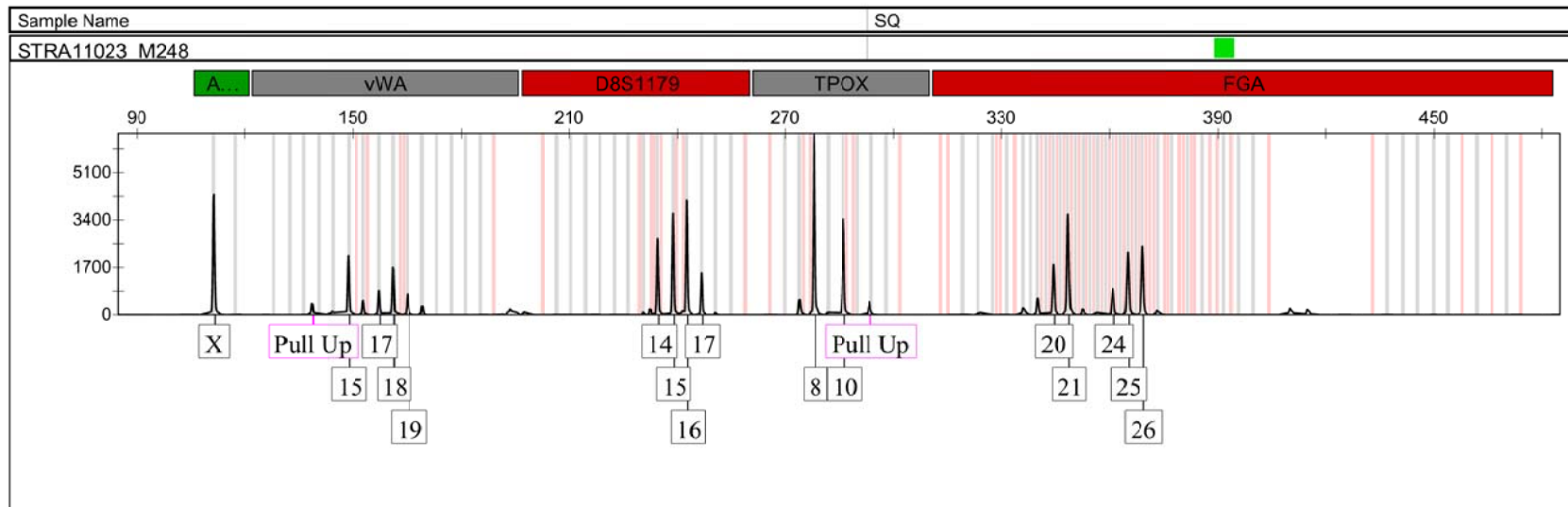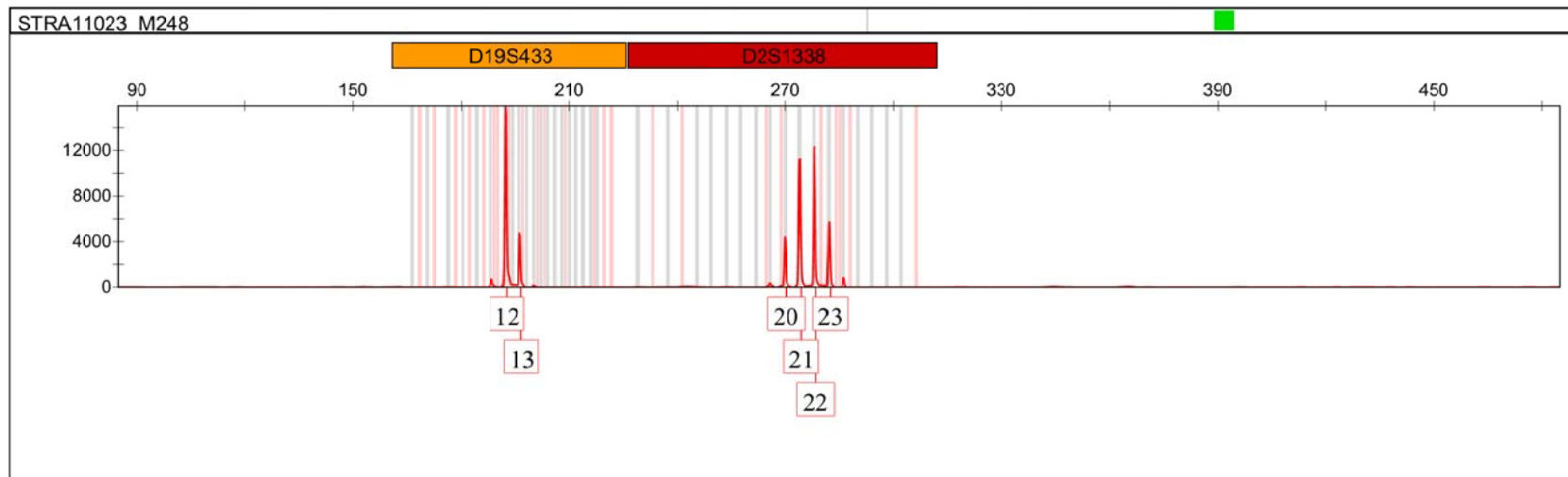

Supplement: S3 File — Short Tandem Repeat (STR) analysis of the A2780.m248 derived cell line used in these studies. (PDF) [file pone.0219006.s006.pdf]
