# Supplementary material for: Preservation of cellular nano-architecture by the process of chemical fixation for nanopathology
Source: PLoS One. 2019 Jul 22;14(7):e0219006. doi: 10.1371/journal.pone.0219006 (PMC6645510; doi:10.1371/journal.pone.0219006)
Supplement: S1 File — Supplementary information file with additional details and explanation of the PWS system, acquisition and analysis procedures, and the meaning of Σ. (DOCX) [file pone.0219006.s004.docx]

Supplementary Information

Preservation of cellular nano-architecture by the process of chemical fixation for nanopathology

Xiang Zhou, Scott Gladstein, Luay M. Almassalha, Yue Li, Adam Eshein, Lusik Cherkezyan, Parvathi Viswanathan, Hariharan Subramanian, Igal Szleifer, Vadim Backman

*PWS System, Acquisition, and Analysis*

The PWS system is built into a Leica DMIRB microscope. In summary, white light from an LED source (X-Cite 120LED) is passed into the system, focused onto the cells through a high NA oil immersion objective (63x Leica HCX PL APO, NA 1.4 or 0.6), the backscattered light is collected and passed through a spectral filter (Cri VariSpec LCTF) before imaging with a CCD camera (Hamamatsu Image-EM CCD). Live-cell PWS microscopy measures the optical interference signal of the backscattering light, which is produced by 1) a strong reflection from the cell-glass interface due to the large refractive index mismatch (referred to as the reference), and 2) the spatial variations of refractive index within the cell due to the organization of cellular structure. These two signals interfere producing spectral variations that vary based on the nanoscale organization of the cellular structure.

To acquire a PWS measurement, wide-field monochromatic microscopy images are acquired at every wavelength between 500 to 700 nm using the spectral filter and stored in a three-dimensional data cube I(x, y, λ), where x and y are the spatial location within a 2D image and λ is wavelength. PWS measurements are collected for every cell as well as a blank region of the dish to normalize our lamp spectra and isolate spectral variations due to interference. After spectral normalization, Σ is calculated as the standard deviations of the spectral variations (λ) at each location in the image. Σ is a measurement of the heterogeneity of macromolecular density with a sensitivity to length scales between 20-350 nm. It is proportional to key metrics of nanoscale organization: the standard deviation of the spatial variations of molecular density ($\delta\rho$) and either the molecular correlation length or the scaling exponent of the mass of chromatin (*D*): $\Sigma\propto\left( D-D_{0} \right)\delta\rho$, where *D*_0_~1.5 (1, 2). $\delta\rho$ is related to the volume fraction of molecular crowders $\varphi$: $\delta\rho\propto\sqrt{\left( 1-\varphi\right)\varphi}$. *D* characterizes the type of scaling between the mass of chromatin (*M*) contained within a sphere of radius *R*: $M\propto R^{D}$; *D*<3 for fractal scaling (in which case *D* is referred to as the mass fractal dimension), which is typical for polymeric structures such as chromatin.

In detail, if an inhomogeneous sample with a refractive index distribution of n_1_[1+n_Δ_(r)] as a function of distance r is placed into the focal plane of a PWS microscope, then the spectral variance of the image intensity, $\Sigma^{2}(x,y)$, within the measured wavenumber range, Δ*k*, will be related to the organization of refractive index within the sample as

$$\frac{E\left[ \Sigma^{2} \right]}{k_{c}L}=\frac{Rk_{c}}{\Delta k}\int_{T_{3D}} \Phi_{n_{\Delta}}\left( k \right)d^{3}k\propto\sigma_{n_{\Delta}}^{2}l_{c}$$

, where $E\left[ \Sigma^{2} \right]$ is the expected value of $\Sigma^{2}$_,_ k_c_ is the central wavenumber of the illumination bandwidth evaluated inside the sample, L is the sample thickness, R=R_012_T_01_T_10_ is an intensity normalization based on the transmission (T_01_, T_10_) and reflection (R_01_) intensities at the sample interfaces, T_3D_ includes spatial frequencies k with longitudinal coordinates k_z_ between 2k_1_ and 2k_2_ (k_1_ and k_2_ are the lower and upper wavenumbers of the instrument bandwidth) evaluated within the sample, $\Phi_{n_{\Delta}}=\left| \mathcal{F}\left\{ n_{\Delta}\left( r \right)/V \right\} \right|^{2}$ is the power spectral density of the sample RI variations n_Δ_ normalized by sample volume V, σ_nΔ_ is the standard deviation of RI fluctuations, and l_c_ is the RI correlation length (1, 2).

**References**

1. Cherkezyan L, Zhang D, Subramanian H, Capoglu I, Taflove A, Backman V. Review of interferometric spectroscopy of scattered light for the quantification of subdiffractional structure of biomaterials. BIOMEDO. 2017;22(3):030901-.

2. Cherkezyan L, Capoglu I, Subramanian H, Rogers JD, Damania D, Taflove A, et al. Interferometric spectroscopy of scattered light can quantify the statistics of subdiffractional refractive-index fluctuations. Phys Rev Lett. 2013;111.
